# Multicenter real-life evaluation of the Post-CAR prognostic index for patients with large B-cell lymphoma after CAR-T failure

**DOI:** 10.1186/s13045-025-01771-6

**Published:** 2026-01-08

**Authors:** Mirko Farina, Elena Maiolo, Chiara Ghiggi, Piera Angelillo, Mattia Novo, Francesca Maria Quaglia, Luana Schiattone, Marta Lisa Battista, Lara Mannelli, Valeria Tomarchio, Elisa Lucchini, Luca Pagliaro, Greta Scapinello, Miriam Marangon, Sabrina Pelliccia, Roberta Sciarra, Domenico Russo, Francesco Zaja

**Affiliations:** 1 Unit of Blood Diseases and Bone Marrow Transplantation, Cell Therapies and Hematology Research Program, Department of Clinical and Experimental Science, ASST- Spedali Civili di Brescia, University of Brescia, Brescia, 25123 Italy; 2 Department of Laboratory and Infectious Sciences, Fondazione Policlinico Universitario A. Gemelli IRCCS, Rome, Italy; 3 Hematology and Cellular Therapies Unit, IRCCS Ospedale Policlinico San Martino, Genoa, Italy; 4 IRCCS San Raffaele Scientific Institute, Milan, Italy; 5 Division of Hematology, AOU Città Della Salute e Della Scienza di Torino, Torino, Italy; 6 Unit of Hematology, DAI Medico Generale, AOUI Verona, Verona, Italy; 7 Unit of Hematology, S. Spirito Civil Hospital, Pescara, Italy; 8 Unit of Hematology and Bone Marrow Transplantation, S. Maria della Misericordia Hospital, Azienda Sanitaria Universitaria Friuli Centrale, Udine, Italy; 9 Division of Oncohematology and Clinical Hematology, ASL Toscana Centro, S. Stefano Hospital, Prato, Italy; 10 Operative Research Unit of Hematology, Fondazione Policlinico Universitario Campus Bio-Medico, Rome, Italy; 11 Hematology Unit, Azienda Sanitaria Universitaria Giuliano-Isontina, Trieste, Italy; 12 Department of Medicine and Surgery, University of Parma, Parma, Italy; 13 Hematology Unit, Department of Medicine, University Hospital of Padua, Padua, Italy; 14 Oncohematology, Hematopoietic Transplantation and Cellular Therapies Unit, IRCCS Centro di Riferimento Oncologico, Aviano, Italy; 15 Hematology Unit, Department of Clinical and Molecular Medicine, Sant’Andrea University Hospital, Sapienza University of Rome, Rome, Italy; 16 Division of Hematology, Fondazione IRCCS Policlinico San Matteo, Pavia, Italy; 17 Department of Medical, Surgical and Health Sciences, University of Trieste, Trieste, Italy

**Keywords:** Post CAR-T failure, PC-PI score, Large B-cell lymphoma, Overall survival

## Abstract

Most patients with large B-cell lymphoma (LBCL) progressing after CAR-T therapy experience poor survival and lack standardized treatment strategies. Prognostic tools are needed to guide decision-making at relapse. The Post-CAR Prognostic Index (PC-PI), recently proposed by Iacoboni et al., combines five routine clinical variables to stratify outcomes after CAR-T failure: ECOG (> 0), hemoglobin (< 10 g/dL), LDH (≥ 2xULN), number of extranodal sites (> 1) and time from CAR-T to progression (< 4 months). We evaluated the PC-PI in a retrospective multicenter study including 125 LBCL patients relapsing or refractory after axicabtagene-ciloleucel or tisagenlecleucel, treated between 2019 and 2023 across 16 Italian centers belonging to the Fondazione Italiana Linfomi network. Median overall survival (OS) was 4.9 months, with 6- and 12-month OS rates of 44.9% and 28.5%, respectively. The PC-PI discriminated prognosis effectively: high-risk patients had a median OS of 1.8 months, intermediate-high 2.2, intermediate-low 8.7, while in the low-risk group median OS was not reached (p<0.0001). Results remained consistent after excluding patients receiving only palliative care. Post-progression therapy markedly influenced survival: patients receiving active treatment achieved a median OS of 7.3 months versus 0.7 without further therapy (p<0.0001). Bispecific antibodies conferred the best outcomes (HR 0.44, p=0.02), with 6- and 12-month OS rates of 90% and 55%. Our findings confirm the prognostic value of the PC-PI and support its use in clinical practice as a tool for risk-adapted management of LBCL after CAR-T failure.

**Supplementary Information:**

The online version contains supplementary material available at 10.1186/s13045-025-01771-6.

To the editor,

Chimeric antigen receptor (CAR) T-cell therapy has transformed the treatment of relapsed/refractory large B-cell lymphoma (LBCL) [1–3]. Nonetheless, approximately 60% relapse or progress, a condition associated with limited survival and scarce evidence-based guidance [4–6]. In this scenario, clinicians must weigh aggressive salvage options against toxicity, while considering timely palliative care. Identifying prognostic factors for post-CAR-T progression is crucial to guide treatment intensity and improve quality of life [7–9]. The Post-CAR Prognostic Index (PC-PI) by Iacoboni et al. [10], represents a relevant and pragmatic step toward risk-adapted decision-making. The PC-PI combines five routine clinical variables at CAR-T progression or relapse—ECOG performance status, lactate dehydrogenase (LDH), hemoglobin < 10 g/dL, number of extranodal sites, and time from CAR-T infusion to progression—to classify patients into distinct risk groups with markedly different outcomes.

We qualitatively validated the PC-PI in a retrospective multicenter study including 125 LBCL patients progressing after axicabtagene ciloleucel (axi-cel) or tisagenlecleucel (tisa-cel) received in their third or later line of therapy between 2019 and December 31, 2023, across 16 Italian centers of the Fondazione Italiana Linfomi network. Institutional review board approval was obtained before data collection, and all procedures were conducted in accordance with the Declaration of Helsinki; written informed consent was available for all participants.

Of the 255 patients who received CAR-T therapy, 125 (49%) [65 axi-cel, 60 tisa-cel] subsequently relapsed or were refractory (Table 1). At the time of progression, the median age was 60.2 years (IQR 57.8–67.8). Most patients had advanced-stage disease (stage III–IV in 76%), with a median of 1 extranodal site (IQR 0–2). Median time from CAR T-cell infusion to relapse/progression was 2.2 months (IQR 1.2–4.2) and median follow-up from CAR-T failure was 20.4 months (95%CI, 14.0–24.1). Relapse or progression was histologically confirmed in 50% of cases, with CD19 loss detected by immunohistochemistry in 26 of 51 (51%) patients tested. The first subsequent treatment included immunotherapy or targeted agents in 57% of patients (20/125 with bispecific antibodies [BiABs]), chemotherapy or radiotherapy in 21%, and palliative care in 22% (Supplementary Material).

Our data confirm the prognostic value of the PC-PI [10] to effectively stratify outcomes in patients following CAR-T therapy failure. Median overall survival (OS) of the entire cohort was 4.9 months (95%CI 2.99–6.89), with a 6- and 12-month OS of 44.9% and 28.5%, respectively. Outcomes varied significantly by PC-PI groups: high-risk patients had a median OS of 1.8 months (95%CI, 0.99–3.38), intermediate-high risk 2.2 months (95%CI, 1.51–4.93), intermediate-low 8.7 months (95%CI, 5.59–17.28), while in the low-risk group the median OS was not reached (95%CI, 11.17–NA) (*p* < 0.0001, Fig. 1). Post-progression therapy had a major impact on survival, with patients receiving active treatment achieving a median OS of 7.3 months (95%CI, 5.2–9.5) versus 0.7 months (95%CI, 0.4–1.5) in those receiving no further therapy (*p* < 0.0001). The predictive performance of the PC-PI remained robust even after excluding patients who received only palliative care (Fig. 1B), and the score retained its ability to distinguish high- and low-risk patients irrespective of the CAR-T product used (Supplementary Material).

Patients treated after CAR-T failure with BiABs had significantly improved outcomes compared with those receiving other therapies (HR, 0.44; 95% CI, 0.21–0.88; *p* = 0.02), with 6- and 12-month OS rates of 90% and 55%, respectively. The PC-PI also effectively stratified high- vs. low-risk patients among those treated with BiABs or other immunotherapies (Supplementary Material).

A main strength of our analysis is its multicenter design, involving a larger number of centers than the study by Iacoboni et al. [10] and including both high- and low-volume CAR-T institutions. This confirms that the PC-PI is reproducible and applicable across heterogeneous real-world settings. Importantly, we show that the PC-PI retains its prognostic value regardless of the CAR-T product used and remains predictive among patients treated with BiABs or other immunotherapies after CAR-T failure. We acknowledge the retrospective design and treatment heterogeneity as limitations; however, the consistent prognostic discrimination across all subgroups supports the reliability and applicability of the PC-PI.

In conclusion, this multicenter real-world evaluation supports the PC-PI as a robust and clinically useful tool for risk stratification after CAR-T failure. Its reproducibility across diverse centers and therapeutic settings underlines its potential for integration into future risk-adapted post–CAR-T management strategies.

**Fig. 1:**
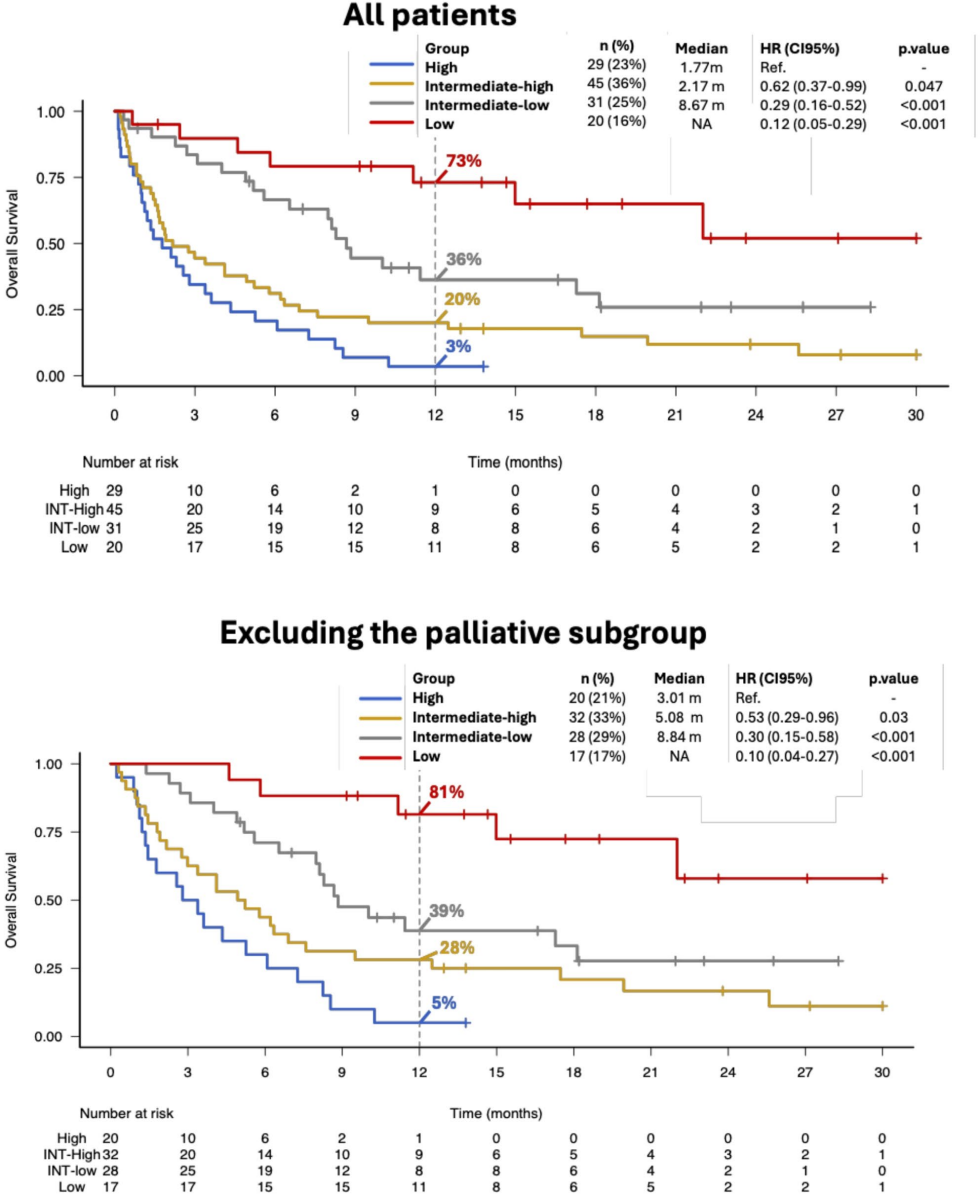
Overall survival in the entire cohort and in the subset excluding patients receiving palliative treatment

**Table 1:** Patient characteristics overall and according to PC-PI risk class

Variables	All patients *N* = 125	High *N* = 29	Int-high *N* = 45	Int-low *N* = 31	Low *N* = 20
**Age**, *median years (range)*	60.2 (18–75)	59.1 (18–75)	63 (38–75)	61(22–74)	61 (18–73)
**Male sex**, *n (%)*	76 (61)	20 (69)	29 (64)	13 (42)	14 (70)
**Histology**, *n (%)* - *DLBCL* - *HGBL* - *tFL* - *PMBL*	76 (61) 24 (19) 17 (14) 8 (6)	15 (52) 7 (24) 3 (10) 4 (14)	28 (62) 9 (20) 7 (16) 1 (2)	20 (65) 7 (22) 4 (13) 0 (0)	13 (65) 1 (5) 3 (15) 3 (15)
**Prior lines > 2**, *n (%)#*	34 (27)	8 (28)	13 (29)	8 (26)	5 (25)
**Primary refractory**, n (%)#*	89 (64)	17 (59)	31 (69)	18 (58)	14 (70)
**Previous HCT**, *n (%)#*	22 (18)	3 (10)	7 (16)	4 (13)	8 (40)
**Construct**, *n (%)* - Axi-cel - Tisa-cel	65 (52) 60 (48)	15 (52) 14 (48)	21 (47) 24 (53)	19 (61) 12 (39)	10 (50) 10 (50)
**Best response to CART**, *n (%)* - CR - PR - SD - PD	28 (22) 36 (29) 12 (10) 49 (39)	2 (7) 5 (17) 2 (7) 20 (69)	5 (11) 13 (29) 4 (9) 23 (51)	13 (42) 9 (29) 4 (13) 5 (16)	8 (40) 9 (45) 2 (10) 1 (5)
**CAR T-cell infusion to PD** - Median months (IQR) - < 4 months, n (%)	2.2 (1.23–4.17) 92 (74)	1.2 (0.87–2.1) 28 (97)	2 (1.23–3.23) 40 (89)	3 (1.85–5.73) 19 (61)	6 (4.3–9.7) 5 (25)
**ECOG**, *n (%)* - 0 - 1 - > 1	26 (21) 39 (31) 60 (48)	2 (7) 7 (24) 20 (69)	4 (9) 12 (27) 29 (64)	4 (13) 17 (55) 10 (32)	16 (80) 3 (15) 1 (5)
**Stage**, *n(%)* - I - II - III - IV	11 (9) 19 (15) 26 (21) 69 (55)	1 (3) 1 (3) 5 (17) 22 (76)	3 (7) 9 (20) 9 (20) 24 (53)	3 (10) 6 (19) 6 (19) 16 (52)	4 (20) 3 (15) 6 (30) 7 (35)
**Extranodal sites** - Median (IQR) - *≥* 2, *n (%)*	2 (0–2) 38 (31)	2 (2–4) 21 (72)	1 (0.25–2.25) 14 (31)	1 (0–1) 1 (3)	1 (0–1) 0 (0)
**Hemoglobin** - Median g/dL (IQR) - < 10 g/dL, *n (%)*	10.9 (9.4–12) 33 (29)	10.7(10–11.4.4) 7 (24)	11 (9.1–12) 13 (29)	11(9.5–11.8) 8 (26)	12 (9.8–12.9) 5 (25)
**Neutrophils** - Median x10^9^/L (IQR) - < 1.0 × 10^9^/L, *n (%)*	1,95 (1.1–3.4) 22 (20)	1.65 (1.2–2.7) 4 (14)	3 (1.59–4.18) 6 (13)	1 (0.9–2.3) 7 (23)	2 (1.1–3.3) 5 (25)
**Platelets** - Median x10^9^/L (IQR) - < 50 × 10^9^/L, *n (%)*	116 (72.5–175) 23 (21)	142 (47–190) 7 (25)	125 (84–151) 7 (16)	113 (67–165) 6 (19)	127 (81–179) 3 (15)
**LDH**, *n (%)* - > 1 xULN - *≥* 2 xULN	85 (68) 33 (26)	26 (90) 18 (62)	31 (69) 13 (29)	17 (55) 1 (3)	11 (55) 1 (5)
**Subsequent strategy**, *n (%)* - Immuno/Targeted - Chemo/Radiotherapy - Palliative care	47 (38) 51 (40) 27 (22)	6 (21) 14 (48) 9 (31)	17 (38) 16 (35) 12 (27)	15 (48) 13 (42) 3 (10)	9 (45) 8 (40) 3 (15)
**Allo-HCT**, *n (%)*	19 (15)	0 (0)	5 (11)	10 (32)	4 (20)

DLBCL, diffuse large B-cell lymphoma; HGBL, high-grade B-cell lymphoma; tFL, transformed follicular lymphoma; PMBL, primary mediastinal B-cell lymphoma; CR, complete response; PR, partial response; SD, stable disease; PD, progressive disease; Allo-HCT, allogeneic hematopoietic cell transplantation; IQR, interquartile range; LDH, lactate dehydrogenase; Int-high, intermediate-high risk; Int-low, intermediate-low risk

## Supplementary Information

Supplementary Material 1

## Data Availability

Data are available upon reasonable request to the corresponding author.

## Abbreviations

CAR-T: Chimeric antigen receptor T-cell
CI: Confidence interval
CR: Complete remission
ECOG: Eastern Cooperative Oncology Group Performance Status
HR: Hazard ratio
IQR: Interquartile Range
LBCL: Large B-cell lymphoma
LDH: Lactate dehydrogenase
p: p-value
PC-PI: Post-CAR Prognostic Index
PD: Progressive disease
PR: Partial response
SD: Stable disease
