# Supplementary material for: Multicenter real-life evaluation of the Post-CAR prognostic index for patients with large B-cell lymphoma after CAR-T failure
Source: J Hematol Oncol. 2026 Jan 8;19:4. doi: 10.1186/s13045-025-01771-6 (PMC12784594; doi:10.1186/s13045-025-01771-6)
Supplement: Supplementary file 1 — Supplementary Material 1 [file 13045_2025_1771_MOESM1_ESM.docx]

**Supplementary Material**

**Table 1 Baseline patient characteristics at the time of CAR-T therapy failure by treatment group.** Patients received bispecific antibodies (n = 20), immunotherapy or targeted therapy (n = 27), chemotherapy (n = 21), or a polatuzumab-based regimen (n = 25). Four patients received radiotherapy, but since this is not a systemic treatment and given the small number, they were not included in the table.

|  | **BiABs***  **N=20** | **Immunotherapy/**  **target therapy^§^**  **N=27** | **Chemotherapy**  **N=21** | **Polatuzumab-based terapy**  **N=25** |
| --- | --- | --- | --- | --- |
| **Age**, *median* *years (range)* | *59.9 (41-73)* | *57.0 (18-73)* | *59.5 (18-76)* | *57.2 (22-75)* |
| **Male sex**, *n (%)* | *13 (65)* | *14 (52)* | *17 (81)* | *13 (52)* |
| **Histology**, *n (%)*  *-DLBCL*  *-HGBL*  *-tFL*  *-PMBL* | *11 (55)*  *4 (20)*  *4 (20)*  *1 (5)* | *16 (59)*  *4 (15)*  *3 (11)*  *4 (15)* | *11 (52)*  *6 (28)*  *2 (10)*  *2 (10)* | *17 (68)*  *4 (16)*  *4 (16)*  *0 (0)* |
| **Construct**, *n (%)*  - Axi-cel  - Tisa-cel | *14 (70)*  *6 (30)* | *13 (48)*  *14 (52)* | *8 (38)*  *13 (62)* | *15 (60)*  *10 (40)* |
| **CAR T-cell infusion to PD**  Median months (IQR)  < 4 months, n (%) | *3.3 (2.2-7.1)*  *12 (60)* | *2.2 (1.2-3.5)*  *22 (81)* | *2.07 (1.1-3.5)*  *18 (86)* | *3.03 (1.57-5.7)*  *16 (64)* |
| **ECOG**, *n (%)*   - 0 -1 - > 1 | *13 (65)*  *7 (35)* | *16 (59)*  *11 (41)* | *8 (38)*  *13 (62)* | *17 (68)*  *8 (32)* |
| **Stage**, *n (%)*   - *I-II* - *III-IV* | *7 (35)*  *13 (65)* | *6 (22)*  *21 (78)* | *4 (19)*  *17 (81)* | *8 (32)*  *17 (68)* |
| **PC-PI score,** *n (%)*   - **Low** - **Int-Low** - **Int-high** - **High** | *5 (25)*  *8 (40)*  *3 (15)*  *4 (20)* | *4 (15)*  *7 (26)*  *14 (52)*  *2 (7)* | *2 (10)*  *1 (5)*  *7 (33)*  *11 (52)* | *4 (16)*  *10 (40)*  *8 (32)*  *3 (12)* |

******BiABs: glofitamab in 11 patients (55%) and epcoritamab in 9 patients (45%).*

**^§^***Immunotherapy and target therapy: lenalidomide (14, 52%), tafasitamab-lenalidomide (5, 19%), brentuximab vedotin - nivolumab (3, 11%), vibostolimab-pembrolizumab (2, 7%), iberdomide (2, 7%), ibrutinib (1, 4%).*

***Figure 1 Distribution of PC-PI scores in patients previously treated with Axi-cel and Tisa-cel.***

Overall survival (OS) according to PC-PI score in patients previously treated with Axi-cel (n = 65) and Tisa-cel (n = 60). Despite the smaller sample size, the PC-PI score maintains a clear ability to stratify high- and low-risk survival outcomes (p<0.001 in both the subgroups). The separation between low and intermediate-low risk groups remains significant, whereas the difference between intermediate-high and high-risk groups is attenuated and no longer statistically significant

**

**

***Figure 2 Distribution of PC-PI scores in patients treated with bispecific antibodies (BiABs).***

The PC-PI score allowed prediction of overall survival (OS; 9.7 months vs. not reached) in high-risk (high + intermediate-high) and low-risk (low + intermediate-low) groups. Due to the limited number of patients, low and intermediate-low categories were combined into a single low-risk group, and high and intermediate-high into a single high-risk group. Among treated patients, 11 (55%) received glofitamab and 9 (45%) received epcoritamab.

**

***Figure 3. Distribution of PC-PI scores in patients treated with immunotherapy or targeted therapy (Total = 47; BiABs = 20; other immunotherapy/targeted therapies = 27).*** The PC-PI score discriminated overall survival (OS), with median OS of 6.8 months (95% CI, 1.9–11.4) in the high-risk group (high + intermediate-high) versus not reached (95% CI, 8.1–NA) in the low-risk group (low + intermediate-low). Due to the limited sample size, low and intermediate-low categories were combined into a single low-risk group, and high and intermediate-high categories into a single high-risk group. Treatment regimens other than BiABs included in the immunotherapy/targeted therapy group are listed in Table 2.

**Table 2: Immunotherapy/targeted therapy regimens other than BiABs.**

| **Regimen** | n (%) |
| --- | --- |
| Lenalidomide | 14 (51.9) |
| Tafasitamab–lenalidomide | 5 (18.5) |
| Brentuximab vedotin–nivolumab | 3 (11.1) |
| Vibostolimab–pembrolizumab | 2 (7.4) |
| Iberdomide | 2 (7.4) |
| Ibrutinib | 1 (3.7) |
| Total | 27 (100) |
